# A Combined mRNA- and miRNA-Sequencing Approach Reveals miRNAs as Potential Regulators of the Small Intestinal Transcriptome in Celiac Disease

**DOI:** 10.3390/ijms222111382

**Published:** 2021-10-21

**Authors:** Ineke Luise Tan, Donatella Barisani, Roberto Panceri, Rutger Modderman, Marijn Visschedijk, Rinse K. Weersma, Cisca Wijmenga, Iris Jonkers, Rodrigo Coutinho de Almeida, Sebo Withoff

**Affiliations:** 1 Department of Genetics, University Medical Center Groningen, University of Groningen, 9700 RB Groningen, The Netherlands; i.l.tan@umcg.nl (I.L.T.); r.modderman@umcg.nl (R.M.); c.wijmenga@umcg.nl (C.W.); i.h.jonkers@umcg.nl (I.J.); 2 Department of Gastroenterology and Hepatology, University Medical Center Groningen, University of Groningen, 9700 RB Groningen, The Netherlands; m.c.visschedijk@umcg.nl (M.V.); r.k.weersma@umcg.nl (R.K.W.); 3 School of Medicine and Surgery, University of Milano-Bicocca, 20900 Monza, Italy; donatella.barisani@unimib.it; 4 Fondazione MBBM, S. Gerardo Hospital, 20900 Monza, Italy; r.panceri@asst-monza.it; 5 Section Molecular Epidemiology, Department of Biomedical Data Sciences, Leiden University Medical Center, 2300 RC Leiden, The Netherlands; rodrigocout@gmail.com

**Keywords:** miRNA‒target gene regulation, post-translational transcript regulation, autoimmunity

## Abstract

Celiac disease (CeD) is triggered by gluten and results in inflammation and villous atrophy of the small intestine. We aimed to explore the role of miRNA-mediated deregulation of the transcriptome in CeD. Duodenal biopsies of CeD patients (*n* = 33) and control subjects (*n* = 10) were available for miRNA-sequencing, with RNA-sequencing also available for controls (*n* = 5) and CeD (*n* = 6). Differential expression analysis was performed to select CeD-associated miRNAs and genes. MiRNA‒target transcript pairs selected from public databases that also displayed a strong negative expression correlation in the current dataset (R < −0.7) were used to construct a CeD miRNA‒target transcript interaction network. The network includes 2030 miRNA‒target transcript interactions, including 423 experimentally validated pairs. Pathway analysis found that interactions are involved in immune-related pathways (e.g., interferon signaling) and metabolic pathways (e.g., lipid metabolism). The network includes 13 genes previously prioritized to be causally deregulated by CeD-associated genomic variants, including STAT1. CeD-associated miRNAs might play a role in promoting inflammation and decreasing lipid metabolism in the small intestine, thereby contributing unbalanced cell turnover in the intestinal crypt. Some CeD-associated miRNAs deregulate genes that are also affected by genomic CeD-risk variants, adding an additional layer of complexity to the deregulated transcriptome in CeD.

## 1. Introduction

In patients with celiac disease (CeD), immune-mediated destruction of the small intestinal villous structure takes place as a response to the presence of dietary gluten. CeD occurs in 1–2% of the Caucasian population in genetically predisposed individuals [1,2]. Nearly all CeD patients carry the genetic risk human leukocyte antigen (HLA) haplotypes HLA-DQ2 or HLA-DQ8. Besides the HLA region, more than 40 non-HLA CeD risk loci have been identified by genome-wide association studies (GWAS) [3,4].

Upon gluten intake, antigen-presenting cells present deamidated gliadin peptides to CD4+ cells in the context of the predisposing HLA-types (HLA-DQ2/8). The activated CD4+ gluten-specific T-cells subsequently stimulate CD8+ T-cells, which eventually move to the intraepithelial compartment and destroy the villous structure of the small intestine [1,5].

Previous studies have shown that the transcriptome of the small intestine is affected in CeD [6,7,8]. Affected pathways include immune-related pathways for B- and T-cells and metabolic pathways (e.g., fatty-acid metabolism). A recent study showed that CeD-associated genomic variants (single nucleotide polymorphisms (SNPs)) influence gene expression and compiled a list of 118 prioritized genes likely to play a role in CeD pathophysiology [9]. This list includes genes involved in immune cell migration, activation, and differentiation. In addition to SNPs, gene expression can be regulated by multiple other factors, both on the transcriptional and post-transcriptional level, including microRNAs (miRNAs) [10]. In the current study, we examined the role of small intestinal miRNAs in CeD pathophysiology.

miRNAs are small non-coding RNAs consisting of 19–24 nucleotides. The physiological role of cellular miRNAs is to fine-tune gene expression by directing the RNA-induced silencing complex to target messenger RNAs (mRNAs) [11]. miRNA target recognition takes place via pairing of the seed region of the miRNA (nucleotides 2–7) with the 3′ untranslated region of the target mRNA, which eventually results in inhibition of translation or promotion of decay of the target transcript [11].

Recently, extracellular miRNAs in the circulation have been proposed as biomarkers for CeD [12]. The small intestine could be the source of these circulating miRNAs, as it has been shown that the small intestinal miRNA profiles are different between CeD and controls [13,14,15,16,17]. However, the functional roles of both circulating and intracellular miRNAs in CeD pathophysiology are not yet fully understood. To uncover the functions and pathways in which CeD-associated miRNAs play a role, the transcripts targeted by the deregulated miRNAs need to be identified. One expects that when miRNAs are overexpressed, target transcripts are downregulated [14,17]. It is also important to investigate these interdependencies in a tissue-specific context, as miRNA expression is highly cell type‒specific [10,18,19]. In the context of CeD, only a few miRNA‒transcript pairs have been investigated thus far as no high-throughput studies have searched for such miRNA‒target transcript pairs in CeD [14,17].

To get insight into how miRNAs affect target genes and pathways in the small intestine of CeD patients, we performed miRNA- and RNA-sequencing on small intestinal biopsies from CeD patients and controls. Using these samples, we investigated which miRNAs were inversely correlated with predicted and experimentally validated target transcripts and used these data to construct a miRNA‒transcript interaction network for CeD. Subsequent pathway-analyses on the target genes revealed that CeD-associated miRNAs are associated with increased inflammatory processes and unbalanced cell turnover in the intestinal crypt. Several miRNA target genes revealed by our analysis have previously been shown to be affected by CeD-risk SNPs, adding another level of complexity to the deregulated gene expression in CeD. 

## 2. Results

### 2.1. Differential Expression Analysis in CeD Identifies Immune-Related Genes and miRNAs 

**Figure 1:**
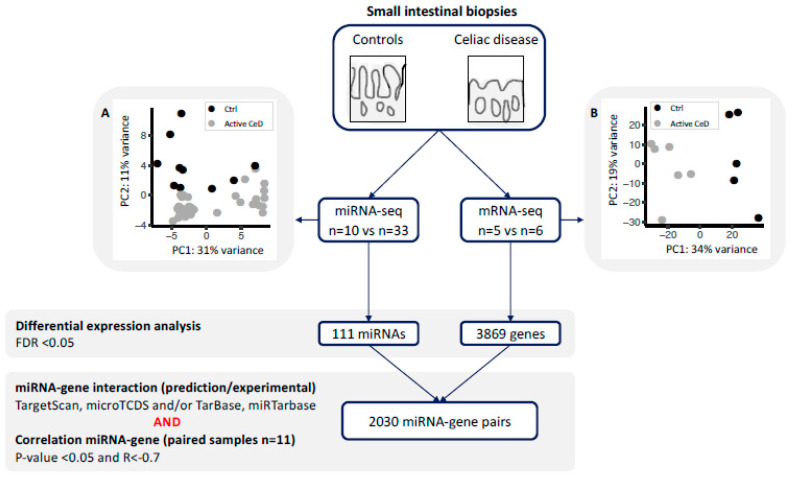
miRNA and gene expression profiles were generated for small-intestinal biopsies using a next generation sequencing approach. Principal component analysis using (**A**) the miRNA-seq profile and (**B**) the mRNA-seq analysis shows clear separation of controls (black) and active celiac disease (CeD) patients (grey). Differential expression analysis was performed between CeD and controls (FDR = *p*-value adjusted for multiple testing). For all the differentially expressed miRNAs and genes, we extracted previously described miRNA-target transcript interactions. In total, 2030 miRNA–transcript target pairs remained with a negative Pearson’s correlation R < −0.7.

To start, we used principal component analysis (PCA) to investigate whether miRNA profile correlates with CeD status. No outliers were observed in the PCA. The PCA showed a clear significant association with disease status (Figure 1A), independent of sex, age, and RNA isolation method.

Differential expression analysis identified 111 miRNAs to be differentially expressed between CeD and controls (FDR < 0.05): 52 that were decreased and 59 that were increased in CeD (Supplementary Table S2). These included miRNAs that had been previously identified to be decreased in CeD: miR-338-5p (Log2 Fold Change (LFC) = −2, FDR = 1.4 × 10^−11^), miR-192-5p (LFC = −1.2, FDR = 1.1 × 10^−8^), miR-194-5p (LFC = –0.8, FDR = 1.2 × 10^−4^), miR-31-3p (LFC = −1.0, FDR = 7.0 × 10^−7^) (Supplemental Table S2) [14,15,20], and ‘immune miRNA’ miR-155-5p (LFC = 1.2, FDR = 1.3 × 10^−5^) [11,21,22,23,24].

No outliers were observed in the mRNA expression profile in a PCA. The mRNA expression profile of CeD biopsies differed significantly from that of control biopsy according to the PCA (Figure 1B), independent of sex, age, and RNA isolation method.

In total, 3869 genes were differentially expressed between CeD and controls (FDR < 0.05), of which 2209 were downregulated and 1660 were upregulated (Supplementary Table S3). We compared this differentially expressed gene list to the list of Loberman-Nachum et al., who previously compiled genes consistently reported to be involved in CeD by multiple studies (Supplementary Table S4) [8]. The majority of genes in the Loberman-Nachum et al. consensus set (*n* = 403) [8] were also concordantly and significantly differentially expressed in our dataset (*n* = 334) (334/403 = 83%). These genes included the five genes that showed the highest discriminatory value between CeD and controls in their dataset: LPL (encoding lipoprotein lipase, plays an important role in lipid clearance, utilization, and storage), BIRC3 (C-IAP-2/baculoviral IAP repeat containing 3, an anti-apoptotic protein binding to TRAF-1 and TRAF-2), UGT2B7 (UDP glucuronosyltransferase family 2 member B7, involved in conjugation and subsequent elimination of potentially toxic compounds), THSD4 (thrombospondin type 1 domain containing 4, a peptidase involved in matrix homeostasis), and HMGCS2 (3-hydroxy-3-methylglutaryl-coA synthase 2, involved in lipid metabolism) [8,25]. The consensus set included genes that are associated with genetic risk loci for CeD, including STAT1 (LFC 1.6, FDR = 2.7 × 10^−13^), which encodes a transcription factor important in the response to interferon signaling [8,9].

To identify pathways related to CeD, we performed pathway-enrichment analysis on the differentially expressed genes in our study. GO terms associated to the down- and up-regulated genes in our study are displayed in Supplementary Figure S1. The transcripts upregulated in CeD are associated with several immune pathways, including B- and T-cell activation (e.g., ‘regulation of lymphocyte activation’ (FDR = 4.6 × 10^−13^) and ‘interferon-gamma pathways’ (FDR = 4.5 × 10^−8^)), and with pathways related to the cell cycle (e.g., ‘positive regulation of cell cycle process’ (FDR = 3.4 × 10^−11^)). Downregulated genes were associated to several metabolic pathways, including lipid metabolism (‘Fatty acid metabolic process’ (FDR = 2.7 × 10^−27^) and ‘digestion’ (FDR = 1.94 × 10^−5^)). These and similar pathways have previously been linked to CeD [8]. 

### 2.2. miRNA‒Target Transcript Interaction Network

**Figure 2:**
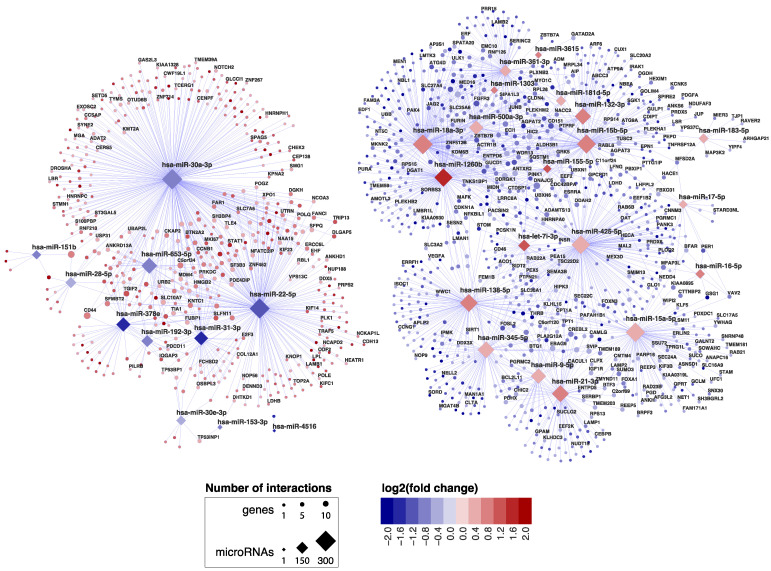
miRNA–target transcript network in small intestinal biopsies. This network shows the 2030 miRNA–target transcript pairs that were selected by the workflow in Figure 1. Only the gene names of experimentally validated target transcripts are shown. Supporting data for the miRNA-mRNA pairs are shown in Supplementary Table S1.

In order to identify miRNAs and their target transcripts, we focused on those miRNAs that were differentially expressed between CeD and controls. We selected miRNA‒target pairs from two prediction tools (TargetScan and microTCDS) and from databases that list experimentally validated miRNA‒target pairs (TarBase and miRTarbase) (Figure 1). Afterwards, we performed a Pearson correlation between the selected miRNAs and genes. This identified 2030 negatively correlated miRNA‒target transcript pairs (R < −0.7 and *p*-value < 0.05). The resulting miRNA‒target transcript interaction network consists of 31 miRNAs connected to 1344 genes (Figure 2, supporting data displayed in Supplementary Table S5). As mentioned before, we identified 3869 genes to be differentially expressed between CeD and controls, 2209 downregulated genes and 1660 upregulated genes. In total, 39% of all downregulated genes (866/2209) and 29% of all upregulated genes (478/1660) are targeted by at least one differentially expressed miRNA. Of the 2030 miRNA‒transcript pairs, only 423 miRNA‒transcript interactions have been experimentally validated. Transcripts that were targeted by more than one miRNA showed a slight, but significantly stronger, negative correlation with miRNA levels compared to genes targeted by only one miRNA (respectively, mean correlation median −0.78 interquartile range (IQR) [−0.75; −0.81] versus correlation −0.76 IQR [−0.73; −0.82], MWU *p*-value = 0.002).

MiRNA families consist of miRNAs that share homology in their seed sequence, resulting in target transcript similarities [11]. In our miRNA‒target transcript network, multiple members of the same miRNA family could be identified amongst both upregulated miRNAs (miR17 family: miR-18a-3p and miR-17-5p; miR15 family: miR-15a/b-5p and miR-16-5p) and downregulated miRNAs (miR30 family: miR-30a/e-3p; miR28 family: miR-151b and miR-28-5p).

### 2.3. Pathway Enrichment Analyses

**Figure 3:**
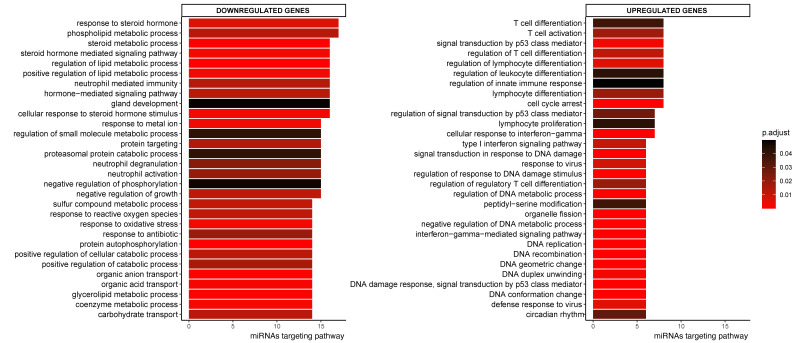
Top 30 enriched GO pathways associated with the down- or upregulated genes in CeD that are targeted by at least one miRNA. Sorted by how many miRNAs targeted the transcripts in the pathway. The colorscale indicates significance of pathway enrichment as indicated with p.adjust.

To assess which pathways might be affected by CeD-associated miRNAs, we performed pathway enrichment analyses based on the transcripts present in the miRNA‒target transcript network. This identified 360 pathways that were significantly associated with the miRNA‒target transcript network (Supplementary Table S6). Figure 3 shows the top 30 pathways associated with the network, split into up- and downregulated transcripts and sorted based on how many different miRNA target transcripts were present in the pathway. Downregulated genes in the miRNA‒target transcript network were associated with pathways related to metabolism, such as lipid metabolism, whereas upregulated genes were associated with immune pathways related to T-cells, response to interferon-gamma, and cell-cycle (Figure 3 and Table S6). 

We prioritized miRNA target transcripts based on the criteria that they are targeted by multiple miRNAs and that there is supporting experimental evidence for the miRNA‒target transcript interaction. This analysis identified 492 transcripts that were targeted by at least two miRNAs, and there is experimental evidence for at least one of the interacting miRNAs for 34% (168/492) of these target transcripts. The downregulated targets (118/168) are significantly enriched with transcripts involved in metabolic pathways (phospholipid metabolic process, FDR = 0.03) and epithelial cell maturation (FDR = 0.046), whereas the upregulated transcripts targets are involved in cell-cycle pathways (e.g., mitotic nuclear division, FDR = 0.003), positive regulation of type I interferon (FDR = 0.03), and the NOTCH pathway (FDR = 0.023).

**Figure 4:**
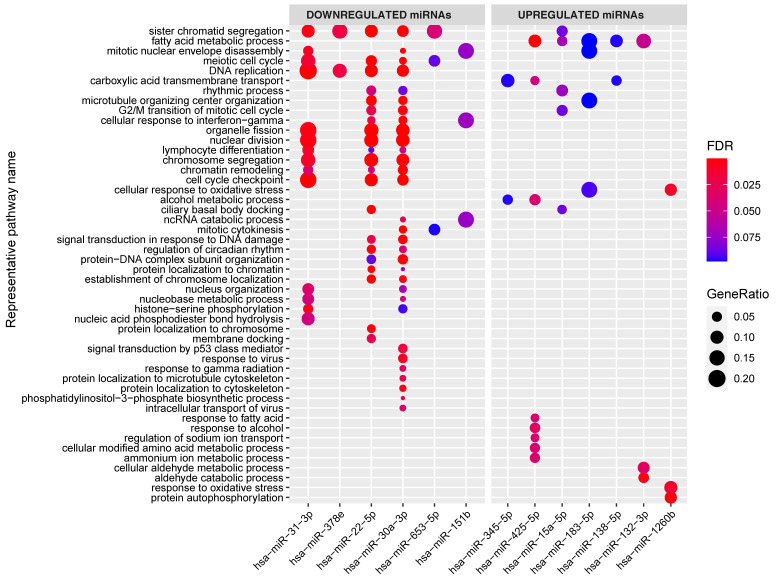
Enrichment of the target genes associated with the individual miRNAs in the miRNA–target transcript network. Y-axis shows representative GO terms that summarize the groups of GO terms selected by REVIGO (input: all the GO terms of the miRNAs combined). miRNAs are sorted from lowest fold change (miR-31) to highest fold change (miR-1260b) in the CeD versus controls comparison. The significance of the enrichment is indicated in FDR by the colorscale and the GeneRatio is indicative of the ratio of genes targeted by the miRNA and the total number of genes in the pathway.

To investigate whether the individual miRNAs in the network regulate specific pathways, we also performed gene set enrichments per miRNA (Figure 4). This revealed that, in our miRNA‒target transcript network, multiple individual miRNAs target similar pathways even though their target transcripts vary. For example, the 44 target transcripts of miR-31-3p (see Figure 4) are significantly enriched in transcripts involved in lymphocyte differentiation, and this was also the case for the target transcripts of miR-22-5p and miR-30a-3p.

### 2.4. Cell Type‒Enrichment Analysis

As small and bulk RNA sequencing results can be influenced by tissue composition, we also assessed the association between the tissue composition of the biopsy, the disease status of the patient, and the miRNA expression.

**Figure 5:**
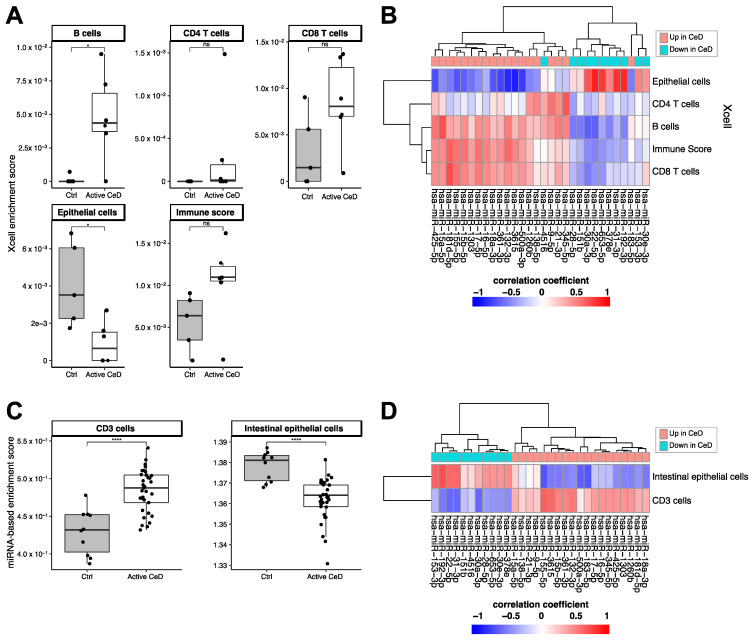
Cell type–enrichment based on Xcell enrichment scores calculated from the RNA-seq data (**A**,**B**) or based on the miRNA based cell type–enrichment score (**C**,**D**). (**A**) Immune cell enrichment scores show an increased trend in CeD. Epithelial cells show a lower enrichment in CeD. (**B**) Heatmap showing the (unscaled) Pearson’s R correlation coefficient between miRNA levels and the cell type–enrichment score. miRNAs that are downregulated in CeD (blue) show a high positive correlation with epithelial cells, whereas upregulated miRNAs show the opposite trend. (**C**) Enrichment for CD3+ associated miRNAs is increased in CeD. Enrichment for epithelial-specific miRNAs is lower in CeD than in controls. (**D**) Heatmap showing the (unscaled) Pearson’s R correlation coefficient between miRNA levels and the enrichment for CD3+ or intestinal epithelial cells. Mann-Whitney U *p*-value: n.s. >0.05, * 0.01–0.05, **** <0.0001.

Using the xCell package, we calculated cell type‒enrichment scores based on the RNA-seq data for multiple cell types relevant in CeD pathophysiology: epithelial cells, CD4- and CD8-positive T-cells, and B-cells (Figure 5A,B). Although the differences between CeD (*n* = 6) and controls (*n* = 5) did not reach significance for any of these cell types, immune cells did show a higher enrichment in CeD biopsies (B-cells: *p* = 0.03; CD8+ T-cells *p* = 0.076; immune cell score *p* = 0.094), and epithelial cells were significantly depleted in CeD compared to controls (*p* = 0.03) (Figure 5A). To investigate in which cell types the miRNAs in the miRNA‒target transcript network are expressed, we correlated the expression levels of the miRNAs with the cell type–enrichment scores. Figure 5B shows that miRNAs downregulated in CeD were correlated with enrichment for epithelial cells and that miRNAs upregulated in CeD were correlated with enrichment for immune cells. 

We then explored whether the CeD miRNAs are expressed in a cell type‒specific manner. A previous overview by De Rie et al. of the enrichment of specific miRNAs in purified human cell types had shown that miRNA expression is highly cell type‒specific [26]. We used the De Rie et al. data to explore whether the 111 up- and downregulated miRNAs in CeD are likely expressed by immune cells or intestinal epithelial cells. Here, we observed a significant correlation between the fold change of miRNAs between CeD and controls in our study and the enrichment scores for CD3+ T-cells (Pearson’s R = 0.27 *p* = 0.02) and intestinal epithelial cells (Pearson’s R = −0.29, *p* = 0.01) reported by De Rie et al. Enrichment scores of the other cell subtypes that were analyzed did not reach significance: CD4+ T-cells (*p* = 0.97), CD8+ T-cells (*p* = 0.59), and B-cells (*p* = 0.894). Because not all the CeD miRNAs we identified were included in De Rie et al., we calculated a miRNA-based enrichment score for each miRNA profile by calculating enrichment for the top 10 CD3+ miRNAs and the top 10 intestinal epithelial cell miRNAs from De Rie et al. Our CeD miRNA profiles showed an enrichment for De Rie et al.’s CD3+ miRNAs and a depletion of De Rie et al.’s intestinal epithelial cell miRNAs (Figure 5C). Moreover, correlation of the miRNA-based enrichment scores with the expression of the miRNAs that were included in our miRNA‒target transcript network led to similar results (Figure 5D). miRNAs downregulated in CeD were enriched for miRNAs expressed in intestinal epithelial cells, whereas miRNAs upregulated in CeD were enriched for the immune cell‒specific miRNAs.

These results suggest that cell type–composition can partially explain the differences in miRNA expression between CeD and controls. Therefore, we again tested the differences between CeD and controls (Supplementary Table S2), including the miRNA-based cell type–enrichment scores in the statistical model to correct for cell type‒composition. Of the 31 miRNAs in the miRNA‒target transcript network, nine showed differences between CeD and controls (FDR < 0.1) that were independent of enrichment of CD3+ or intestinal epithelial cell types.

### 2.5. Linking the miRNA‒Target Transcript Network to Genes in CeD-Associated Genetic Risk Regions 

**Figure 6:**
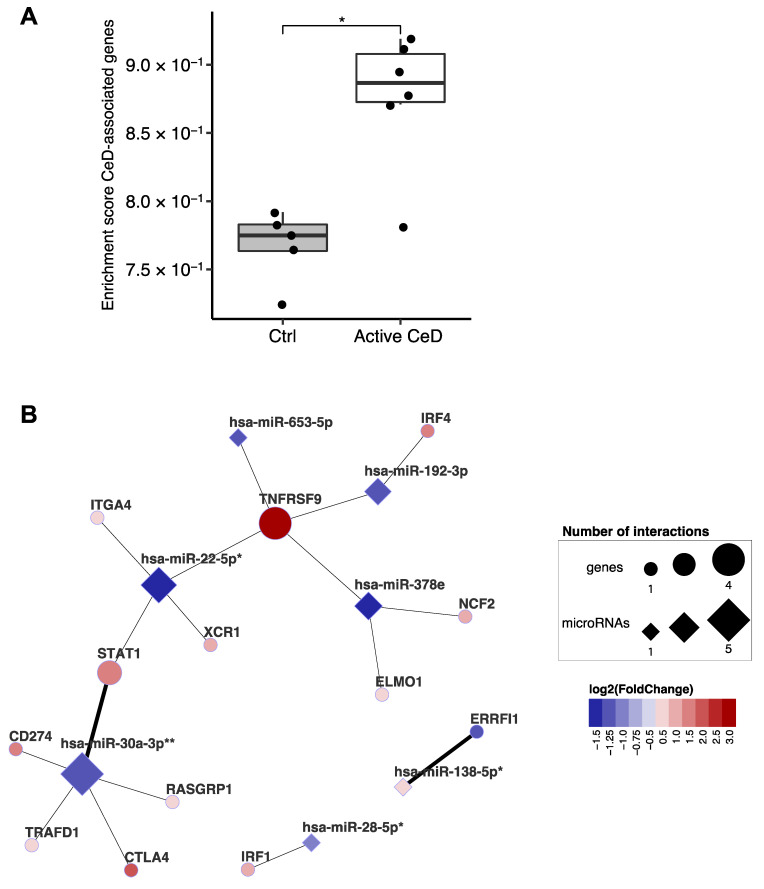
Overlap between genes of which the expression is affected by both celiac disease risk–SNPs and by miRNAs. (**A**) Single sample enrichment analysis reveals that the genes prioritized by van der Graaf et al. are enriched in active CeD compared to controls [9]. This analysis included the 102 genes (out of 118) with sufficient expression in the biopsy transcriptome dataset. Mann-Whitney U *p*-value: * 0.01–0.05. (**B**) Subset of the miRNA-target transcript interaction network. Only genes that overlap with the prioritized CeD genes are displayed. Thick connecting lines indicate that the interaction was previously experimentally validated. After correction for cell type composition (miRNA-based enrichment score for intestinal epithelial cells and CD3+ cells), the miRNAs indicated with asterisk still show a trend between CeD and controls (FDR: * 0.05–0.1, ** <0.05).

A previous study integrated multiple in-silico approaches to prioritize genes in CeD-associated genomic risk regions and identified 118 genes as likely causal in CeD [9]. Our data show a clear enrichment of the transcripts of these prioritized genes in CeD biopsies (Figure 6A). Of the 118 genes, 102 were expressed in our biopsy transcriptome dataset, and 13 of the 102 are also found in our miRNA‒target transcript network (Figure 6B). This suggests that, in addition to being affected by genetic factors that predispose to CeD, these genes might also be regulated post-transcriptionally by CeD-associated miRNAs. This network includes the previously mentioned STAT1 [9]. In the miRNA‒target transcript network, STAT1 is associated with miR-22-5p (R = −0.86; *p* = 6.6 × 10^−4^) and miR-30a-3p (R = −0.88; *p* = 3.9 × 10^−4^), and there is other previous experimental evidence (TarBase: HITS-CLIP) to support the interaction with miR-30a-3p (Supplementary Table S5).

## 3. Discussion

To our knowledge, this is the first study to use next-generation sequencing to generate a CeD-specific miRNA‒target transcript interaction network, thereby providing the first unbiased analysis of miRNAs and their targets in the context of CeD. For our analyses, we used public databases of predicted miRNA‒target transcript pairs or experimentally validated miRNA‒target transcript pairs that showed a strong negative correlation in our expression dataset. This resulted in a network of 2030 miRNA‒target transcript pairs that provides a starting point for understanding the complex relations between miRNAs and target transcript regulation in CeD pathophysiology.

Our analyses show that miRNA target transcripts are involved in many pathways important in CeD pathogenesis. Downregulated transcripts appear to be involved in, for instance, (lipid) metabolism pathways [12], and upregulated (de-repressed) transcripts appear to play a role in cell cycle pathways and immune-pathways (e.g., T- and B-cell-pathways, interferon pathways) [27,28,29]. The network also includes several sets of miRNAs belonging to the same miRNA families (families miR17, miR15, miR30, and miR28). MiRNA families are defined by homology in their seed sequence, resulting in the targeting of the same transcript [11]. Additionally, we identified different miRNAs that target different transcripts in the same pathways, which suggests that miRNAs from different families can cooperate to enhance repression of particular pathway, as previously suggested by others [19,30].

Our results suggest that CeD-associated miRNAs are involved in regulating barrier homeostasis, a process that is affected in CeD [12]. Some miRNAs appear to do this by affecting lipid metabolism in the small intestine. It has been shown, for instance, that mice with a small intestinal DICER-knock out display abnormal absorption and processing of lipids [31,32]. Lipid metabolism is also important in the maintenance of the regenerative capacity of the small intestinal crypt [33,34]. One of the lipid metabolism transcripts targeted in our small-intestinal miRNA‒target transcript network is the transcription factor PRDM16, which is targeted by miR-500a-3p and miR-361-3p. In a murine model, Stine et al. showed that loss of Prdm16 inhibits transcription of many fatty-acid oxidation genes, resulting in villous atrophy of the small intestine, which is a hallmark of CeD [35]. We observed that a number of fatty-acid oxidation genes that are regulated by PRDM16 (CPT1A, ECI1, CD36, ACSL1, ACAA2, and HADH) are also targeted by CeD-associated miRNAs (miR-361-3p, miR-155-5p, miR-15a-5p, miR-18a-3p, miR-425-5p, and miR-138-5p). This shows that deregulated small intestinal miRNAs in CeD patients contribute to villous atrophy by regulating genes related to fatty-acid metabolism. Another miRNA prioritized in our study, miR-31-3p, is downregulated in CeD and has been previously associated with cell cycle and immune pathways [14,15,20]. Tian et al. showed that miR-31-3p is highly expressed in the intestinal epithelial crypt and that it is an important restorative factor in the intestine through maintenance of the homeostasis of cell turnover from the intestinal crypt to the villous tip [36]. MiR-31-3p knockout mice display more severe intestinal inflammation as a response to chemically induced colitis (DSS), and this response can be dampened by administration of miR-31-3p [37]. Other target transcripts that play a role in intestinal epithelial maintenance are RXRA (targeted by the upregulated 155-5p, miR-1260b, miR-132-3p, miR-425-5p, miR-18a-3p, and miR-425-5p; has a key role in retinol signaling in the differentiation of mature enterocytes [38]), VAV2 (targeted by 155-5p, miR-15a/b-5p, and miR-17-5p; has a role in wound repair in the intestine and in differentiation and migration of mature enterocytes along the crypt-villous axis via RAC1 [39,40]), CUX1 (miR-132-3p; transcription factor targeting VAV2 [40]), and PACSIN2 (miR-155-5p, miR-1260b, miR-138-5p, and miR-361-3p; controls morphology of the microvilli [41]).

MiRNAs also appear to deregulate the immune response in CeD, for instance by affecting interferon signaling, which is key in CeD pathophysiology [9,28,29]. Above, we discussed how miR-31-3p is involved in intestinal barrier homeostasis, but downregulation of miR-31-3p has also been shown to enhance the response of CD8+ cells to viral triggers, leading to a higher pro-inflammatory interferon response [42]. The target genes of miR-31-3p include HMGB2 and PRKDC, which are both nucleic acid sensors that are important in eliciting an interferon response after sensing of cytosolic DNA [43,44]. Interestingly, a paralog of HMGB2, HMGB1, has been proposed as a biomarker for CeD [45,46,47]. Another prioritized and upregulated miRNA is miR-155-5p, a well-described immune miRNA that also has a role in enhancing the interferon response [21,22,23,24]. UBXN1 is a target gene of miR-155-5p in our miRNA‒target transcript network. A previous study has shown that UBXN1 inhibits pro-inflammatory NF-κB signaling and the interferon response to viral stimuli [48,49]. Furthermore, knockdown of the miR-155-5p target gene JUNB in regulatory T-cells has been shown to lead to increased production of pro-inflammatory cytokines, such as IFN-gamma, in colonic tissue [50,51]. Regulatory T-cell function has previously been shown to be affected in individuals with CeD [28]. In addition to miR-155-5p and miR-31-3p, other miRNAs also play a role in interferon signaling. Our network connects six additional downregulated miRNAs (miR-192-3p, miR-30a-3p, miR-653-5p, miR-22-5p, miR-28-5p, and miR-151b) to transcripts involved in interferon-gamma response. Two of these miRNAs, miR-22-5p and miR-30a-3p, were previously described to enhance interferon signaling [52,53]. Altogether, our analysis shows that the deregulated interferon response in CeD is partially regulated by a CeD-specific miRNA profile.

Interestingly, 13 of the CeD-associated target transcripts identified by our network have previously been described to be potentially causally deregulated by CeD-associated SNPs [9]. Two transcripts have also been experimentally validated to be the target of the CeD-associated miRNAs in our network: STAT1 has been shown to be regulated by miR-30a-3p and ERRFI1 by miR-138-5p [9]. MiR-22-5p and miR-30a-3p, which we described above as regulating genes associated with interferon signaling, also affect the transcripts of genes in genetic risk loci for CeD such as STAT1 (miR-22-5p and miR-30a-3p) and TRAFD1 (miR-30a-3p) [9]. Several CeD-associated SNPs can cause altered binding sites for miRNAs, thereby affecting the binding between miRNA and gene, depending on genotype [54]. The current study could not detect these kinds of differences due to the lack of genotype information and, more importantly, limited sample size. However, taken together, these results suggest that miRNAs and genetic risk SNPs associated with CeD cooperate in deregulating the expression of transcripts in CeD pathophysiology.

A limitation in miRNA research is that most miRNA‒target transcript interactions have been predicted by target-prediction algorithms but have not been validated experimentally. In the current network, 423 out of 2030 interactions have experimental evidence supporting these interactions. The best way to get insight into physiological miRNA‒target transcript interactions would be via crosslinking-based methods such as HITS-CLIP, in which miRNAs are crosslinked to target transcripts and subsequently sequenced [11,55]. Both miRNAs and mRNA are expressed in a cell type‒specific manner, and therefore expression in a biopsy is expected to depend on cell type differences between CeD and controls (e.g., expansion of lymphocytes). Indeed, we observed that the predicted cell type composition explained part of the miRNA differences that we observed between CeD and controls. However, even though miRNAs may not be produced in the same cells as the target transcripts, these miRNAs might still be able to affect transcripts with different target cells [56,57]. Because of these cell type‒dependent expression levels, miRNA‒target transcript interactions should ideally be performed at single cell‒level [58,59]. While the currently available single cell techniques are not yet capable of capturing the full spectrum of miRNA‒target transcript interactions, they do hold potential for the future [60]. 

Interestingly, our biopsy-focused approach identified three miRNAs—miR-21-3p, miR-500a-3p, and miR-15b-5p—that also can be detected in circulation as biomarkers for CeD [13,15,16,61] (Tan et al. submitted manuscript). We previously observed that these three miRNAs were upregulated in the circulation of CeD patients compared to controls up to two years the rise in current serological antibodies (anti-tissue transglutaminase) could be detected (Tan et al. submitted manuscript).

Taken together, the results of our paired miRNA‒target transcript sequencing study of small intestinal biopsies of CeD patients versus controls have revealed that the miRNAs deregulated in CeD could play a role in metabolic, cell cycle, and immune pathways that are deregulated in the small intestine in CeD. Our study is an exploratory, hypothesis generating study to investigate the potential role of miRNAs in CeD. Future functional studies should be performed to validate and confirm the role of miRNAs candidates in the pathogenesis of CeD, preferably in a cell-type specific manner. Moreover, to get more insights in the specificity of these regulatory miRNA-gene interaction pathways, it would be of value to also include other types of intestinal inflammation (e.g., Crohn’s disease) in future studies. A better understanding of the role of these miRNAs in CeD pathogenesis could aid in the search for biomarkers relevant to disease processes and the identification of novel therapeutic options for CeD.

## 4. Materials and Methods

### 4.1. Sample Collection

Pediatric patients and controls were included at the San Gerardo Hospital, Monza, Italy. The parents of all participants provided informed consent for the study. Duodenal biopsies were collected from untreated CeD patients at time of diagnosis (*n* = 33). CeD diagnosis was established based on serology (anti-transglutaminase antibodies) and histopathological examination (villous atrophy and influx of intraepithelial lymphocytes). Biopsies were also collected from control individuals (*n* = 10) who underwent upper endoscopies for other indications and did not show signs of CeD in the histopathological examination of small-intestinal biopsies. Clinical characteristics are described in Supplementary Table S1. The study was conducted after approval of the San Gerardo Hospital Ethical Committee, Monza, Italy.

### 4.2. RNA Isolation, Small RNA, and mRNA-Sequencing

RNA was isolated from the small-intestinal biopsies using either the miRVana isolation Kit (Ambion, Carlsbad, CA, USA) or qiazol lysis reagent (Qiagen, Hilden, Germany, 79306). The proportion of CeD and controls did not differ between both isolation methods (χ2 P = 0.43). RNA quality was assessed using the Caliper GX bioanalyzer (Agilent, Santa Clara, CA, USA). Small RNA-libraries were generated starting from 500 ng total RNA using the TruSeq Small RNA Sample Prep kit (Illumina, San Diego, CA, USA), implementing 15 amplification cycles. RNA libraries were prepared as previously described in Zorro et al. [9,62], using the Illumina TruSeq stranded total RNA library kit with a riboZero rRNA depletion step. After measurement of the cDNA concentration, libraries were pooled equimolarly per lane on a HiSeq 2500 (Illumina San Diego, CA, USA). Raw reads were aligned to miRbase 22 (small-RNA-seq) and human_g1k_v37 ensemble Release 75 (RNA-seq), as described previously (Tan et al. submitted manuscript; [62]). For all 43 samples, small-RNA libraries were generated and sequenced. Bulk RNA-sequencing was performed for 5/10 of the controls and 6/33 of the CeD patients (GEO accession number GSE146190) [9,62].

### 4.3. Prioritizing Genes Targeted by miRNAs in the Small Intestinal Biopsies

#### 4.3.1. Differential Expression Analyses in miRNA Sequencing and RNA Sequencing

To find miRNAs and genes that are deregulated in CeD in small intestinal biopsies, we performed differential expression analysis between CeD patients and controls for the miRNA profiles and RNA profiles separately (R-package DESeq2, version 1.26.0 [63]), while correcting for the covariates age and sex. *p*-values were adjusted for multiple testing using the Benjamini-Hochberg correction for False Discovery Rate (FDR) [64]. Regularized log-normalized miRNA and RNA counts were used in all downstream analyses.

#### 4.3.2. miRNA‒Target Transcript Network

To build a miRNA‒target transcript network (see Figure 1), we first identified miRNA‒target transcript pairs by combining data provided by two prediction databases (TargetScan version 7.2 [65] and microTCDS version 7.0 [66]) and by two experimentally-validated miRNA target databases (TarBase version 7.0 [67] and miRTarbase version 7.0 [68]). We then calculated Pearson’s correlations for all miRNA‒target transcript pairs, and only those pairs with negative Pearson’s correlations (R < −0.7) and a *p*-value < 0.05 were subsequently integrated into the miRNA‒target transcript network and visualized with the RedeR package [69]. 

### 4.4. Enrichment Analyses

We performed pathway analyses to identify which pathways were associated to (1) transcripts that were differentially expressed in CeD versus controls and (2) all the transcripts combined that are targeted by miRNAs in the miRNA‒target transcript network. To zoom in on specific functions of the individual miRNAs in the miRNA‒target transcript network, we also performed pathway analyses using separate transcript lists per miRNA. Pathway analyses were performed using the R-package clusterProfiler (version 3.14.3) using Gene Ontology (GO) terms (Biological Process) [70]. The online tool REVIGO was used to reduce the number of redundant GO terms from the long lists of significantly associated GO terms (settings: small 0.5) [71].

### 4.5. Cell Type‒Enrichment Analyses

To gain insight into the cell types that might contribute to the miRNA and bulk transcript differences between CeD and controls, we calculated enrichment scores for cell types using two approaches. In the first approach, we used xCell, an in-silico method that can be used to reliably calculate enrichment of certain cell types in bulk RNA-seq samples [72,73,74]. In the second approach, we used cell type–specific miRNA information to calculate cell type–enrichment scores from the miRNA-seq data. Here, we extracted the top 10 most-enriched miRNAs specific for immune cells and intestinal epithelial cells from a publicly accessible miRNA expression atlas [26]. This list was then used as input for the R-package GSVA (version 1.34.0) to perform single sample enrichment analyses to calculate: (1) the miRNA-based cell type enrichment score and (2) the enrichment for previously prioritized CeD genes [9].

## Data Availability

GEO accession number GSE146190) [9,62].

## Supplementary Materials

The following are available online at https://www.mdpi.com/article/10.3390/ijms222111382/s1.
