# Supplementary figures and images for: A Combined mRNA- and miRNA-Sequencing Approach Reveals miRNAs as Potential Regulators of the Small Intestinal Transcriptome in Celiac Disease
Source: Int J Mol Sci. 2021 Oct 21;22(21):11382. doi: 10.3390/ijms222111382 (PMC8583991; doi:10.3390/ijms222111382)

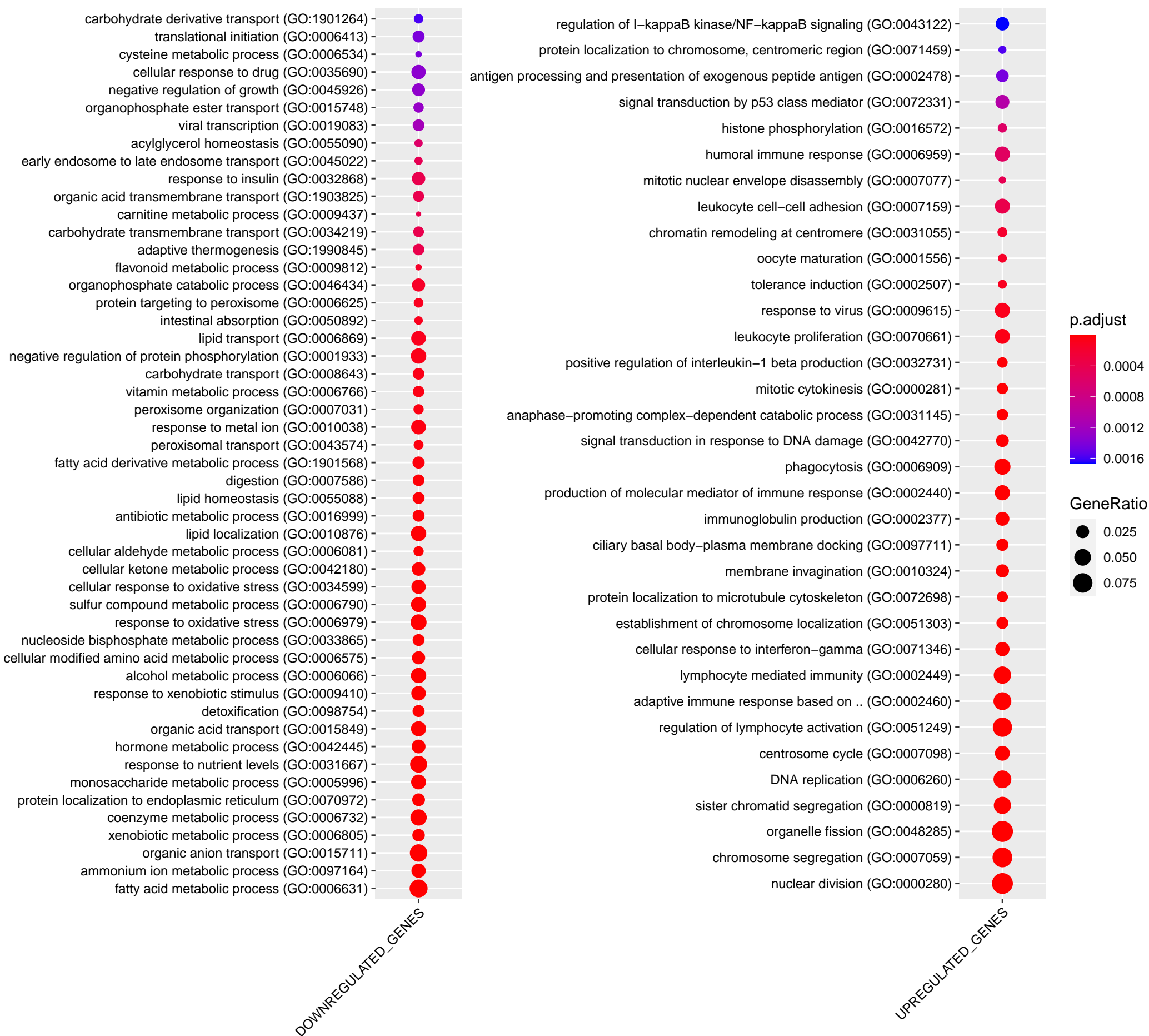

Supplement: Supplementary file 1 [file ijms-22-11382-s001.zip › Supplementary Figure S1.pdf]
